# Application of data pooling to longitudinal studies of early post-traumatic stress disorder (PTSD): the International Consortium to Predict PTSD (ICPP) project

**DOI:** 10.1080/20008198.2018.1476442

**Published:** 2018-06-14

**Authors:** Wei Qi, Andrew Ratanatharathorn, Martin Gevonden, Richard Bryant, Douglas Delahanty, Yutaka Matsuoka, Miranda Olff, Terri deRoon-Cassini, Ulrich Schnyder, Soraya Seedat, Eugene Laska, Ronald C. Kessler, Karestan Koenen, Arieh Shalev, The ICPP, Yael Errera-Ankri, Sara Freednam, Jessie L. Frijling, J. Carel Goslings, Saskia B.J. Koch, Johannes S.K. Luitse, Alexander Mcfarlane, Joanne Mouthaan, Daisuke Nishi, Meaghan O’Donnell, Mark Rusch, Marit Sijbrandij, Derrick Silove, Sharain Suliman, Dick J. Veltman, Mirjam van Zuiden

**Affiliations:** a Department of Psychiatry, New York University School of Medicine, New York, NY, USA; b Department of Epidemiology, Columbia University Mailman School of Public Health, New York , USA; c Faculty of Science, University of New South Wales School of Psychology, Kensington, Australia; d Department of Psychological Sciences, Kent State University, Kent, OH, USA; e Division of Health Care Research, National Cancer Center, Center for Public Health Sciences, Tokyo, Japan; f Department of Psychiatry, University of Amsterdam Academic Medical Center, Amsterdam-Zuidoost, The Netherlands; g Department of Surgery, Medical College of Wisconsin, Milwaukee, WI, USA; h Department of Psychiatry, University of Zurich School of Medicine, Zurich, Switzerland; i Department of Psychiatry, Stellenbosch University School of Medicine and Health Sciences, Cape Town, South Africa; j Department of Health Care Policy, Harvard Medical School, Boston, MA, USA; k Department of Epidemiology, Harvard University T.H. Chan School of Public Health, Boston, MA, USA

**Keywords:** PTSD, pooled analysis, data harmonization, longitudinal, prediction, TEPT, análisis combinado, armonización de datos, longitudinal, predicción, PTSD，汇总分析，数据统一，纵向预测, • This article describes the main methods and challenges of pooling data from 13 different studies of early PTSD (totalling over 6000 subjects), to overcome the limitations of single studies to find predictors of PTSD.• Studies used various inclusion criteria and assessment tools.• One cannot infer general PTSD prevalence from a single study.• Pooling data requires careful data processing with content expertise. It is an important step towards generalizable prediction models for PTSD.

## Abstract

**Background**: Understanding the development of post-traumatic stress disorder (PTSD) is a precondition for efficient risk assessment and prevention planning. Studies to date have been site and sample specific. Towards developing generalizable models of PTSD development and prediction, the International Consortium to Predict PTSD (ICPP) compiled data from 13 longitudinal, acute-care based PTSD studies performed in six different countries.

**Objective**: The objectives of this study were to describe the ICPP’s approach to data pooling and harmonization, and present cross-study descriptive results informing the longitudinal course of PTSD after acute trauma.

**Methods**: Item-level data from 13 longitudinal studies of adult civilian trauma survivors were collected. Constructs (e.g. PTSD, depression), measures (questions or scales), and time variables (days from trauma) were identified and harmonized, and those with inconsistent coding (e.g. education, lifetime trauma exposure) were recoded. Administered in 11 studies, the Clinician Administered PTSD Scale (CAPS) emerged as the main measure of PTSD diagnosis and severity.

**Results**: The pooled data set included 6254 subjects (39.9% female). Studies’ average retention rate was 87.0% (range 49.1–93.5%). Participants’ baseline assessments took place within 2 months of trauma exposure. Follow-up durations ranged from 188 to 1110 days. Reflecting studies’ inclusion criteria, the prevalence of baseline PTSD differed significantly between studies (range 3.1–61.6%), and similar differences were observed in subsequent assessments (4.3–38.2% and 3.8–27.0% for second and third assessments, respectively).

**Conclusion**: Pooling data from independently collected studies requires careful curation of individual data sets for extracting and optimizing informative commonalities. However, it is an important step towards developing robust and generalizable prediction models for PTSD and can exceed findings of single studies. The large differences in prevalence of PTSD longitudinally cautions against using any individual study to infer trauma outcome. The multiplicity of instruments used in individual studies emphasizes the need for common data elements in future studies.

## Abstract (es)

**Antecedentes**: Comprender el desarrollo del trastorno de estrés postraumático (TEPT) es una condición previa para una evaluación de riesgos y una planificación de prevención eficientes. Los estudios hasta la fecha han sido específicos del sitio y de la muestra. Hacia el desarrollo de modelos generalizables de desarrollo y predicción de TEPT, el Consorcio Internacional para Predecir el TEPT (ICPP) recopiló datos de 13 estudios de TEPT longitudinales basados en la atención aguda realizados en seis países diferentes.

**Objetivo**: describir el enfoque del ICPP para la combinación de datos y la armonización, y presentar los resultados descriptivos entre estudios que informan el curso longitudinal del trastorno de estrés postraumático después del trauma agudo.

**Métodos**: se recogieron datos a nivel de ítem de 13 estudios longitudinales de adultos sobrevivientes de trauma civil. Se identificaron y armonizaron constructos (por ejemplo, trastorno de estrés postraumático, depresión), medidas (preguntas o escalas) y variables de tiempo (días desde el trauma) y se recodificaron aquellas con codificación inconsistente (por ejemplo, educación, exposición de trauma a lo largo de la vida). Administrado en 11 estudios, la Escala de TEPT Administrada por el Clínico (CAPS) surgió como la principal medida del diagnóstico y la gravedad del TEPT.

**Resultados**: el conjunto de datos agrupado incluyó 6254 sujetos (39,9% mujeres). La tasa promedio de retención de estudios fue 87.0% (rango: 49.1% a 93.5%). Las evaluaciones iniciales de los participantes se realizaron dentro de los dos meses posteriores a la exposición al trauma. Las duraciones de seguimiento variaron de 188 a 1110 días. Reflejando los criterios de inclusión de los estudios, la prevalencia de TEPT basal difirió significativamente entre los estudios (rango: 3,1% a 61,6%), y se observaron diferencias similares en las evaluaciones posteriores (respectivamente, 4,3% a 38,2% y 3,8% a 27,0% para la segunda y tercera evaluaciones).

**Conclusión**: la agrupación de datos de los estudios recopilados de forma independiente requiere una cuidadosa conservación de los conjuntos de datos individuales para extraer y optimizar las comunalidades informativas. Sin embargo, es un paso importante hacia el desarrollo de un modelo de predicción robusto y generalizable para el TEPT y poder superar los hallazgos de estudios únicos. Las grandes diferencias en la prevalencia de TEPT advierten longitudinalmente contra el uso de cualquier estudio individual para inferir el resultado del trauma. La multiplicidad de instrumentos utilizados en estudios individuales enfatiza la necesidad de elementos de datos comunes en estudios futuros.

## Abstract (zh)

**背景**：了解创伤后应激障碍（PTSD）的发展是高效进行风险评估和制定预防计划的先决条件。 迄今为止的研究一直是针对特定地区和样本的。 旨在开发PTSD发展和预测的一般化模型，国际预防创伤后应激障碍联合会（ICPP）汇编了来自在六个不同国家进行的13项纵向急性护理PTSD研究的数据。

**目标**：描述ICPP的数据汇集和同一的方法，并展示跨研究描述性结果，说明急性创伤后PTSD的纵向过程。

**方法**：收集了13个成人平民创伤幸存者纵向研究的题目数据。 确定并统一了心理结构（如PTSD，抑郁），测量方式（问题或量表）和时间变量（创伤后天数），并对编码不一致的患者（例如教育，终身创伤暴露）进行重新编码。 在11项研究中，《临床使用的创伤后应激障碍量表（CAPS）》成为PTSD诊断和严重程度的主要指标。

**结果**：汇总的数据集包括6254个受试者（39.9％的女性）。 研究的平均保留率为87.0％（范围：49.1％至93.5％）。 参与者的基线评估在创伤暴露的两个月内。 随访时间从188天到1110天不等。 研究的纳入标准、基线PTSD的患病率在研究间存在显着差异（范围：3.1％至61.6％），在随后的评估中存在类似的差异（第二和第三次评估范围分别为4.3％至38.2％，3.8％至27.0％）。

**结论**：从独立收集的研究汇集数据需要仔细管理各个数据集以提取和优化信息共同性。 然而，这是发展PTSD有力且可推广的预测模型迈出的重要一步，并且可能超过单一研究结果的价值。 PTSD纵向患病率的巨大差异警告不能使用任何单一研究来推断创伤结果。 个别研究中使用的多种测量强调了未来研究中对共同数据元素的需求。

## 1. Introduction

Post-traumatic stress disorder (PTSD) is the most frequent and best documented psychopathological consequence of traumatic events. PTSD is tenacious, debilitating, and treatment refractory in many cases (Breslau, Peterson, Poisson, Schultz, & Lucia, 2004; Hoskins et al., 2015; Institute of Medicine, 2014; Kessler, 2000; Roberts, Roberts, Jones, & Bisson, 2015, 2016; Schnyder et al., 2015; Sijbrandij, Kleiboer, Bisson, Barbui, & Cuijpers, 2015). Early interventions may reduce the prevalence of chronic PTSD among survivors at risk (Kearns, Ressler, Zatzick, & Rothbaum, 2012), but they are resource demanding and effective in only a subset of survivors. The frequent presence of spontaneously remitting early symptoms (Bryant et al., 2015; Galatzer-Levy et al., 2013) makes it difficult to differentiate those at risk for chronic disorder from those who will remit on their own. This, in turn, constitutes a barrier to targeting prevention efforts to those at risk. Improving PTSD prediction is a highly desirable clinical and public health goal.

Acute care centres are a reliable source of recently traumatized survivors. Emergency departments in the USA, for example, evaluate over 30 million individuals with traumatic injuries every year (Bergen and National Center for Health Statistics (U.S.), 2008; Bonnie, Fulco, and Liverman, 1999; Cougle, Kiplatrick, and Resnick, 2012; Kilpatrick et al., 2013; McCaig, 1994; National Center for Injury Prevention, 2012; Rice, MacKenzie, Jones, and Associates, 1989); the prevalence of PTSD following acute care admissions is similar to that seen in survivors who are not brought to medical attention (Ameratunga, Tin, Coverdale, Connor, & Norton, 2009; Golding, 1999; Lipsky, Field, Caetano, & Larkin, 2005). PTSD longitudinal trajectories stabilize at 9–12 months after trauma exposure (Kessler, Sonnega, Bromet, Hughes, & Nelson, 1995), with symptoms at 9 and 12 months equally predicting symptoms at 2 and 6 years (Bryant, O’Donnell, Creamer, McFarlane, & Silove, 2013; Shalev et al., 2012; Shalev, Ankri, Peleg, Israeli-Shalev, & Freedman, 2011). Studying PTSD in acute care settings provides an opportunity to follow exposed individuals early on after traumatic events and therefore offers valuable longitudinal information difficult to obtain elsewhere.

Acute-care based studies to date have identified several predictors of PTSD that are accessible in the early aftermath of trauma exposure, including lifetime trauma exposure, disadvantaged socio-demographic background, event severity, and early PTSD symptoms (Ehlers, Mayou, & Bryant, 2003; Freedman, Brandes, Peri, & Shalev, 1999; Karstoft, Galatzer-Levy, Statnikov, Li, & Shalev, 2015; Schnyder, Moergeli, Klaghofer, & Buddeberg, 2001; Shah & Vaccarino, 2015; Shalev, Freedman, Peri, Brandes, & Sahar, 1997; Shalev, Peri, Canetti, & Schreiber, 1996). However, differences in the design, location, sampling, measurements, and length of follow-up of these studies has precluded the development of a generalizable predictive model (Bryant, Creamer, O’Donnell, Silove, & McFarlane, 2012; Bryant et al., 2015; Ehlers, Mayou, & Bryant, 1998; Heron-Delaney, Kenardy, Charlton, & Matsuoka, 2013; Schnyder et al., 2001).

Nonetheless, significant amounts of acute-care based, longitudinal data have been collected to date. Studies (reviewed below) have typically annotated the type of traumatic event, participants’ symptoms, and information about known PTSD predictors, such as gender, lifetime trauma exposure, prior mental illness, education, and recovery environment (Brewin, Andrews, & Valentine, 2000; Bryant et al., 2012; Freedman et al., 2002; Gabert-Quillen et al., 2012; Koren, Arnon, & Klein, 1999; Macklin et al., 1998; Ozer, Best, Lipsey, & Weiss, 2003), to evaluate prediction of non-remitting PTSD. These data constitute a viable source for inferring risk estimates across different studies, while reflecting the specific culture and context in which each study was conducted.

Pooled analysis, otherwise known as individual participant data meta-analysis (Debray et al., 2015), is therefore preferable to conventional meta-analysis, a central-tendency driven quantitative review of published results, in that the latter cannot properly account for cultural and contextual factors, such as samples’ heterogeneities, collection rules, and assumptions underlying the original investigators’ statistical analyses. Inconsistency in the variables included in predictive models and analytic techniques across studies may lead to inaccurate estimates of the results and the conclusions that are drawn. For example, a traditional meta-analysis that examines female gender as a risk factor for PTSD may overlook the effect of explicit and hidden covariates in each study (e.g. exclusion of comorbid disorders or prior PTSD), thereby creating unaccounted for heterogeneity in gender-effect estimates. Pooled data analyses differ from meta-analytic methods in that they rely on studies’ raw data and consequently can reveal the full distribution of variables instead of mean results. They further account for the original studies’ designs and address data analytic heterogeneities (Blettner, Sauerbrei, Schlehofer, Scheuchenpflug, & Friedenreich, 1999). Data pooling is increasingly being used to build large data sets from separately collected samples to reach statistical power and generalizability (e.g. Logue et al., 2015).

Pooling data from different sources, however, requires intense data management, careful identification of constructs and related measures, quality appraisal, and harmonization. Investigators involved in pooling data must carefully consider many important aspects of the studies, including sampling, measurements and assessment schedules, and loss to follow-up. They must also define the dimensions and resolutions within which the ‘pooled’ data set can be reliably interrogated. The steps to conduct pooled analysis include: (1) defining each pooled study’s objectives and inclusion criteria; (2) identifying qualified studies and collecting item-level individual data; (3) harmonizing and merging data from different sources; (4) examining heterogeneity between studies; and (5) analysing pooled data, including sensitivity analyses (Friedenreich, 1993; Smith-Warner et al., 2006).

The International Consortium to Predict PTSD (ICPP) is an effort sponsored by the US National Institute of Mental Health to create a consortium of principal investigators of published and unpublished longitudinal PTSD studies (Bonne et al., 2001; Bryant, Creamer, O’Donnell, Silove, & McFarlane, 2008; deRoon-Cassini, Mancini, Rusch, & Bonanno, 2010; Hepp et al., 2008; Irish et al., 2008; Jenewein, Wittmann, Moergeli, Creutzig, & Schnyder, 2009; Matsuoka et al., 2009a; Mouthaan et al., 2014; Shalev et al., 2012, 2000, 2008; van Zuiden et al., 2017), combine their individual- and item-level data towards carrying out a pooled secondary analysis, and synthesize information about the predictors of PTSD. The ICPP’s goal is to pool and harmonize extant data sets so as to inform PTSD pathogenesis and prediction across trauma types, severity, geography, and clinical circumstances. Participating investigators contributed raw data stripped of personal identification information from current and previous studies. Data sets were reviewed, annotated, harmonized, and used to build a common data set.

This paper describes the ICPP’s approach to pooling PTSD-specific studies, outlines challenges and solutions, describes the data set generated, and presents a descriptive map of longitudinal PTSD research. In the light of our experience, we discuss study-specific and generic aspects of data pooling and analytics.

## 2. Methods

### 2.1. Data sources

Longitudinal studies tracking the development of PTSD among survivors admitted to acute care centres were identified by a literature review and by contacting researchers active in the field. Studies were eligible for inclusion if they (1) evaluated civilian survivors of a distinct traumatic event, (2) had a baseline assessment shortly after trauma exposure, (3) included at least one consecutive assessment of PTSD and PTSD symptoms using validated instruments, and (4) had individual participant data available for pooling.

We contacted 12 principal investigators, whose longitudinal studies evaluated 7737 recent trauma survivors. Ten investigators (6648 participants) agreed to share their data. Investigators provided preliminary descriptions of their studies, studies’ assessment schedules, published results and, when available, codebooks linking data-set items to instruments and measurements. They contributed a total of 16 studies. One study (*N *= 99) was discarded owing to loss of follow-up data, and two others (*N* = 168 and 127) were excluded for lack of item-level data. The studies not included comprised motor vehicle accident (MVA) survivors (100% compared with 77.8% in those included). Table 1 shows the main features of the 13 studies that were included in the final pool. Two longitudinal studies included early interventions. The Jerusalem Trauma Outreach and Prevention Study (JTOPS) had 296 out of 1996 participants randomly assigned to treatment groups that included cognitive behavioural therapy, escitalopram, and placebo (Shalev et al., 2012). The Amsterdam oxytocin study (van Zuiden et al., 2017) evaluated the preventive effect of oxytocin, and data included in the ICPP consisted of that study’s placebo group. Beyond available information regarding general study design, investigators have used various inclusion and exclusion criteria for recruitment (Appendix 1).

**Table 1.** Description of studies in the International Consortium to Predict PTSD (ICPP).

Study ^a^	Principal investigator	Start year	End year	Country	Sample size	Setting	PTSD diagnostic scale	Recruitment	Last follow-up	Number of PTSD assessments	Follow-up rate (%)^b^
Zurich ICU (Hepp et al., 2008)	Schnyder	1995	1997	Switzerland	121	ICU	CAPS	In hospital	3 years	4	90.1
Hadassah startle (Shalev et al., 2000)	Shalev	1995	1998	Israel	235	ER	CAPS	1 week	4 months	2	88.1
Jerusalem fMRI (Bonne et al., 2001)	Shalev	1997	1998	Israel	50	ER	CAPS	1 week	6 months	1	68.0
Zurich ward (Jenewein et al., 2009)	Schnyder	1999	2000	Switzerland	323	Trauma ward	CAPS	In hospital	6 months	2	89.8
Midwest resilience (deRoon-Cassini et al., 2010)	deRoon-Cassini	2000	2003	USA	328	Trauma ward	PDS	In hospital	6 months	3	83.8
Ohio MVA (Irish et al., 2008)	Delahanty	2000	2004	USA	406	Trauma ward	CAPS	In hospital	1 year	3	84.0
Multisite ASD (Bryant et al., 2008)	Bryant	2004	2006	Australia	1084	ER	CAPS	In hospital	1 year	3	88.8
Hadassah cortisol (Shalev et al., 2008)	Shalev	2005	2006	Israel	279	ER	CAPS	1 week	4 months	2	81.6
JTOPS (Shalev et al., 2012)	Shalev	2003	2007	Israel	1996	ER	CAPS	1 week	3 years	4	93.5
TCOM (Matsuoka et al., 2009a)	Matsuoka	2004	2008	Japan	312	CCU	CAPS	In hospital	1.5 years	4	69.9
Amsterdam cortisol (Mouthaan et al., 2014)	Olff	2005	2009	Netherlands	852	ER	CAPS	A few days	1 year	4	89.1
Milwaukee^c^	deRoon-Cassini	2007	2014	USA	214	Trauma ward	PSS	In hospital	2 years	4	49.1
Amsterdam oxytocin (van Zuiden et al., 2017)	Olff	2012	2015	Netherlands	54	ER	CAPS	A few days	6 months	4	85.2

^a ^Studies are listed by chronological order of the end year, from earliest to most recent studies. If two studies ended in the same year, alphabetical order of the principal investigator’s last name is used. If both study end year and principal investigator are the same, the study with a larger sample size is listed first.

^b^ Follow-up rate is defined by the percentage of subjects with at least one follow-up after baseline assessment, using dates of follow-up as an indicator of being assessed. The Midwest resilience study has missing dates for both follow-up time-points. Therefore, the follow-up rate of this study is the percentage of subjects with at least one PTSD assessment after baseline, using the PDS data.

^c ^Unpublished at the time of data transfer.

PTSD, post-traumatic stress disorder; ICU, intensive care unit; fMRI, functional magnetic resonance imaging; MVA, motor vehicle accident; ASD, acute stress disorder; JTOPS, Jerusalem Trauma Outreach and Prevention Study; TCOM, Tachikawa Cohort of Motor Vehicle Accident; ER, emergency room; CCU, critical care centre; CAPS, Clinician Administered PTSD Scale (Blake et al., 1998); PDS, PTSD Diagnostic Scale (Foa, 1995); PSS, PTSD Symptom Scale (Foa & Tolin, 2000).

### 2.2. Constructs and related measures

Following studies’ acceptance, the original data sets were reviewed, individual items were identified and linked with specific instruments (e.g. rating scales), and the latter were mapped into six overarching psychopathological constructs: PTSD and PTSD symptoms, other Diagnostic and Statistical Manual of Mental Disorders, 4th Edition (DSM-IV) Axis I disorders, acute stress symptoms, depression and anxiety symptoms, substance use disorders, and global functioning (Table 2).

**Table 2.** Major constructs measured and instruments used in the International Consortium to Predict PTSD (ICPP).^a^

Constructs	Instruments/questions	Study													Total number of studies
		Zurich ICU (Hepp et al., 2008)	Hadassah startle (Shalev et al., 2000)	Jerusalem fMRI (Bonne et al., 2001)	Zurich ward (Jenewein et al., 2009)	Midwest resilience (deRoon-Cassini et al., 2010)	Ohio MVA (Irish et al., 2008)	Multisite ASD (Bryant et al., 2008)	Hadassah cortisol (Shalev et al., 2008)	JTOPS (Shalev et al., 2012)	TCOM (Matsuoka et al., 2009a)	Amsterdam cortisol (Mouthaan et al., 2014)	Milwaukee^b^	Amsterdam oxytocin (van Zuiden et al., 2017)	
PTSD symptoms	CAPS	4	2	1	2		3	3	2	4	4	4		3	11
	IES/IES-R	3	3	2			2		3		7	5		4	8
	MISS		2						2						2
	PSS					3				7					2
	PDS												4		1
	DTS				2										1
Acute stress responses	PDEQ		1	2	1	1	1	1	3		1	1	1		10
	PDI							1	2		2	1		1	5
	ASDI					1	1	1							3
Depression and anxiety symptoms	HADS	2						3			7	5		4	5
	BDI		3	2					3	4					4
	CESD					3	1						3		3
	STAI		3	2					3						3
Substance use	Smoking						5		1		7	3		1	5
	Alcohol use			1		4	3				7				4
	AUDIT							3				3			2
Other DSM diagnosis	SCID		2	1					2	4					4
	MINI							3			4	5		3	4
Functioning	WHOQOL							3		4		2	3		4
	SF-36					3	1				4				3

^a ^Numbers in the scale × study cells represent the number of times when the specific instrument/question was used to assess participants.

^b ^Unpublished at the time of data transfer.

PTSD, post-traumatic stress disorder; ICU, intensive care unit; MVA, motor vehicle accident; JTOPS, Jerusalem Trauma Outreach and Prevention Study; TCOM, Tachikawa Cohort of Motor Vehicle Accident; ASD, acute stress disorder; DSM, Diagnostic and Statistical Manual of Mental Disorders; CAPS, Clinician Administered PTSD Scale (Blake et al., 1998); IES, Impact of Event Scale (Horowitz, Wilner, & Alvarez, 1979); IES-R, Impact of Event Scale – Revised (Weiss, 2007); MISS, Mississippi PTSD Scale (Vreven, Gudanowski, King, & King, 1995); PDS, PTSD Diagnostic Scale (Foa, 1995); PSS, PTSD Symptom Scale (Foa & Tolin, 2000); DTS, Davidson Trauma Scale (Davidson et al., 1997); PDEQ, Peritraumatic Dissociative Experiences Questionnaire (Marmar, Weiss, & Metzler, 1997); PDI, Peritraumatic Distress Inventory (Brunet et al., 2001); ASDI, Acute Stress Disorder Structured Interview (Bryant, Harvey, Dang, & Sackville, 1998); HADS, Hospital Anxiety and Depression Scale (Zigmond & Snaith, 1983); BDI, Beck Depression Inventory (Beck, Steer, & Brown, 1996); CESD, Center for Epidemiologic Studies Depression Scale (Radloff, 1977); STAI, State–Trait Anxiety Inventory (Spielberger, Gorsuch, & Lushene, 1970); AUDIT, Alcohol Use Disorders Identification Test (Saunders, Aasland, Babor, De La Fuente, & Grant, 1993); SCID, Structured Clinical Interview for DSM Disorders (First, Spitzer, Gibbon, & Williams, 1997); MINI, Mini-International Neuropsychiatric Interview (Lecrubier et al., 1997); WHOQOL, World Health Organization Quality of Life (Kuyken et al., 1995); SF-36, 36-item Short Form Health Survey (Ware & Sherbourne, 1992).

### 2.3. Data harmonization

#### 2.3.1. Participants’ identities and time anchors

To enable data amalgamation, each participant in each of the original studies was assigned an ICPP global identifier. Data in different formats and languages were translated and transformed into a standard format following the procedure described in Appendix 2. Because data represented in these studies involved repeated assessments, a ‘days since trauma’ variable was attached to each instrument, representing the exact timing of the instrument’s administration relative to the traumatic event. Days since trauma were subsequently used to build a master summary sheet including all instances of repeated evaluation. The global ID and the days since trauma in the summary sheet were then used as unique identifiers to link different instruments assessed at different times.

### 2.4. Recoding of variables

#### 2.4.1. Demographics and personal variables

Demographics and baseline information are critical data descriptors and potential predictors (Breslau et al., 1998; Brewin et al., 2000; Karstoft et al., 2015). While age and gender could be reliably derived from studies’ data sets, other variables were differentially collected, and had to be recoded for use in the common data set, as follows.

Variability in reporting education stemmed from using different coding systems (e.g. years of schooling vs highest level of education completed) and from different education models in different countries. For example, Switzerland and Australia have an apprenticeship system after grade 9 or 10, while the USA and other countries have a high-school system that continues until grade 12. This diversity, corroborated with ICPP international investigators, was finally reconciled by reducing ‘education’ level into a dichotomous variable describing whether a person has finished high school (or equivalent education level in the country). Marital status was encoded as a dichotomous variable differentiating ‘married or cohabiting’ from ‘unmarried and not cohabiting’.

Several constructs (e.g. PTSD, depression) were consistently measured, whereas others (e.g. coping mechanism, memory) were measured less frequently. Different instruments were used to capture the same construct. Table 2 summarizes the most frequently measured constructs, instruments used, and the frequency of their usage across studies. Other less common instruments were identified; however, these were not considered for pooling at this point. They were inconsistently measured in a minority of studies and therefore were not included in Table 2. As a result, certain baseline predictors known in the literature were not discussed in the current paper (e.g. pain, medication, income, and social support).

Studies typically used four to seven categories of traumatic events varying from one site to another. For example, the JTOPS study (Shalev et al., 2012) had MVAs, work accidents, physical assaults, terrorist attacks, and other traumas as trauma categories, while the Midwest resilience study (deRoon-Cassini et al., 2010) had MVA, assaults, gunshot wounds, stabbings, falls, work accidents, household accidents, snowmobile accidents, and object-fell-on-person accidents. Based on epidemiological studies showing a higher conditional prevalence of PTSD following interpersonal trauma (Benjet et al., 2016), and categorization of the World Mental Health Survey (Karam et al., 2014), we recoded these variable into (1) MVAs, (2) other non-interpersonal trauma (e.g. work or home accidents, falls, and sports accidents), and (3) interpersonal trauma (e.g. assaults, rape and other violence). Inconsistently encoded prior trauma exposures, using study-specific instruments, were similarly recoded into categories known to differentially predict PTSD (Karam et al., 2014): ‘interpersonal trauma’ (e.g. war-related events, physical violence, sexual violence, terror, and kidnapping) and ‘all other events’.

The severity of traumatic events was assessed in some studies directly or indirectly through several different measurements, including Injury Severity Score, Glasgow Coma Score, amnesia, loss of conscious, Abbreviated Injury Score, ad-hoc severity or exposure levels, length of stay in hospital, and pain score. Trauma severity is not included in this paper due to the lack of consistency across the entire pool. However, it can be used in the future for subsets of studies in which a consistent severity score can be reliably derived.

#### 2.4.2. Measures of PTSD

The following instruments were used to infer PTSD diagnosis and severity:

1. Clinician-Administered PTSD Scale (CAPS; used in 11 studies): a structured clinical interview that evaluates the frequency (0–4) and the severity (0–4) of each DSM-IV PTSD symptom criterion, provides a PTSD total severity score (0–136) (Blake et al., 1998), and uses the DSM-IV decision rule to infer the presence of PTSD (Weathers, Ruscio, & Keane, 1999).
2. PTSD Symptom Scale (PSS; two studies): a validated self- or interviewer-administered rating scale that evaluates PTSD symptom criteria severity (0–3) and infers preliminary DSM-IV PTSD diagnosis (Foa, Riggs, Dancu, & Rothbaum, 1993; Foa & Tolin, 2000).
3. PTSD Diagnostic Scale (PDS; one study): the PDS is another validated scale that measures PTSD severity with all 17 symptoms and can be used for preliminary diagnosis. Symptom frequency is rated from 0 to 3 (deRoon-Cassini et al., 2010; Foa, 1995).

PTSD diagnostic status was redetermined based on these instruments using DSM-IV decision rules. To infer the presence of each PTSD diagnostic criterion we used, for CAPS interviews, the recommended threshold of frequency ≥ 1 and intensity ≥ 2 (Weathers et al., 1999), and for PSS and PDS an item score ≥ 2 (Foa, 1995; Foa, Cashman, Jaycox, & Perry, 1997; Foa et al., 1993; Foa & Tolin, 2000). We also accepted direct reporting, as some studies’ symptom criteria were recorded as ‘present’ versus ‘absent’.

However, criterion A (exposure to traumatic event and initial responses),was not explicitly annotated in many studies and impelled us to conclude that subjects’ trauma exposure was implied by their inclusion in each of the studies.

Symptom duration (≥ 1 month required, criterion E) was inconsistently documented and could not be included in our definition of PTSD. Similarly, PTSD criterion F (clinically significant distress or impairment) was absent from more than half of the studies and thus could not be used to sanction the presence or absence of PTSD. To address this shortcoming, we compared the prevalence of PTSD with and without criterion F. Among 1219 cases meeting criteria B, C, and D across all time-points, only 26 (2.1%) of them did not meet criterion F. We consequently refer to participants who meet PTSD symptom criteria (B, C, and D) as having PTSD.

## 3. Results

### 3.1. Study features

The 13 studies that were included by the ICPP reflected data collected longitudinally in the Netherlands, Switzerland, the USA, Israel, Japan, and Australia. The studies’ sample sizes ranged from 50 to 1996. Studies had up to four repeated PTSD assessments, with the time of final assessments ranging between 4 and 36 months after trauma exposure. Studies recruited from a variety of acute trauma settings, most commonly the emergency room, trauma wards, and intensive care units. The average follow-up rate was 87.0% (range 49.1–93.5%) (Table 1).

### 3.2. Baseline characteristics and demographics

Participants included in the ICPP data set (*n *= 6254) were enrolled between the years 1995 and 2014. Baseline demographic information such as age, gender, marital status, education, and trauma types is provided in Table 3. The participants’ mean age was 37.77 years (range 31.25–42.92). The gender distribution was 39.9% female and 60.1% male. The most frequent traumatic event was MVAs (73.8%), followed by other accidents (18.5%) and interpersonal violence (7.7%). Over 60% of individual participants across all studies had experienced another traumatic event before the current trauma.

**Table 3.** Demographics, trauma type, and prior trauma in International Consortium to Predict PTSD (ICPP) pooled data.

Study	Demographics				Trauma type (%)			Prior trauma (%)		
	Age (years), mean ± SD	Female (%)	Married/cohabiting (%)	Completed high-school education (%)	MVA	Other accidents	Interpersonal	No prior trauma	Prior non-interpersonal trauma	Prior interpersonal trauma
Zurich ICU (Hepp et al., 2008)	37.54 ± 13.24	24.8	43.0	86.8	62.0	38.0	0.0	NA		
Hadassah startle (Shalev et al., 2000)	28.77 ± 10.64	51.9	49.3	NA	84.6	7.7	7.7	9.2	23.7	67.1
Jerusalem fMRI (Bonne et al., 2001)	32.66 ± 8.93	54.0	61.2	76.0	78.0	6.0	16.0	4.0	28.0	68.0
Zurich ward (Jenewein et al., 2009)	40.88 ± 12.89	35.3	39.0	80.5	30.0	70.0	0.0	NA		
Midwest resilience (deRoon-Cassini et al., 2010)	40.42 ± 15.79	67.4	31.8	75.9	58.6	15.6	25.8	28.1	27.5	44.3
Ohio MVA (Irish et al., 2008)	38.20 ± 15.79	40.2	38.3	90.0	100.0	0.0	0.0	5.7	45.5	48.9
Multisite ASD (Bryant et al., 2008)	37.94 ± 13.56	26.9	48.8	74.1	65.0	29.5	5.5	13.0	27.5	59.5
Hadassah cortisol (Shalev et al., 2008)	31.25 ± 10.84	44.4	46.7	98.8	82.6	5.8	11.6	23.6	41.8	34.6
JTOPS (Shalev et al., 2012)	36.26 ± 11.98	46.9	50.7	87.1	84.2	6.9	8.9	29.8	35.7	34.5
TCOM (Matsuoka et al., 2009a)	36.49 ± 15.00	22.7	43.3	79.0	100.0	0.0	0.0	24.2	48.4	27.4
Amsterdam cortisol (Mouthaan et al., 2014)	42.92 ± 15.87	35.4	53.8	78.4	65.6	30.2	4.2	12.4	38.2	49.5
Milwaukee^a^	42.85 ± 16.92	31.8	40.2	83.2	39.2	33.8	27.1	40.0	45.0	15.0
Amsterdam oxytocin (van Zuiden et al., 2017)	35.91 ± 13.30	48.1	50.9	72.5	72.2	16.7	11.1	0.0	48.1	51.9
All studies (pooled)	37.77 ± 14.03	39.9	46.4	81.0	73.8	18.5	7.7	18.2	34.5	47.2

^a ^Unpublished at the time of data transfer.

MVA, motor vehicle accident; ICU, intensive care unit; fMRI, functional magnetic resonance imaging; ASD, acute stress disorder; JTOPS, Jerusalem Trauma Outreach and Prevention Study; TCOM, Tachikawa Cohort of Motor Vehicle Accident; NA, not available.

### 3.3. Sampling heterogeneity

Studies differed in inclusion and exclusion criteria (Appendix 1). The main inclusion criteria were related to the seriousness of injury (e.g. minimum injury severity score), the initial response to the event (e.g. DSM-IV PTSD A2 criterion), and symptom expression after the event (e.g. a minimum score on a screening instrument). For example, three studies (Bryant et al., 2012; deRoon-Cassini et al., 2010; Jenewein et al., 2009) had a minimum hospital admission length (24–48 hours), and three studies (Bonne et al., 2001; Shalev et al., 2012; van Zuiden et al., 2017) had a minimum threshold or criterion for initial symptoms. These criteria screened for more severely injured or more symptomatic patients to be included in the studies. The main exclusion criteria included the extent of injury (aiming to exclude patients considered too severely injured to participate, especially patients with head injury), prior mental health problems (aiming to study the onset of new mental health issues), certain event characteristics (e.g. self-harm), and practical or ethical criteria (e.g. incarceration). Given these criteria, each study can be seen as representing a specific subset of the entire trauma population in medical settings.

### 3.4. Assessment time-points

**Figure 1.**
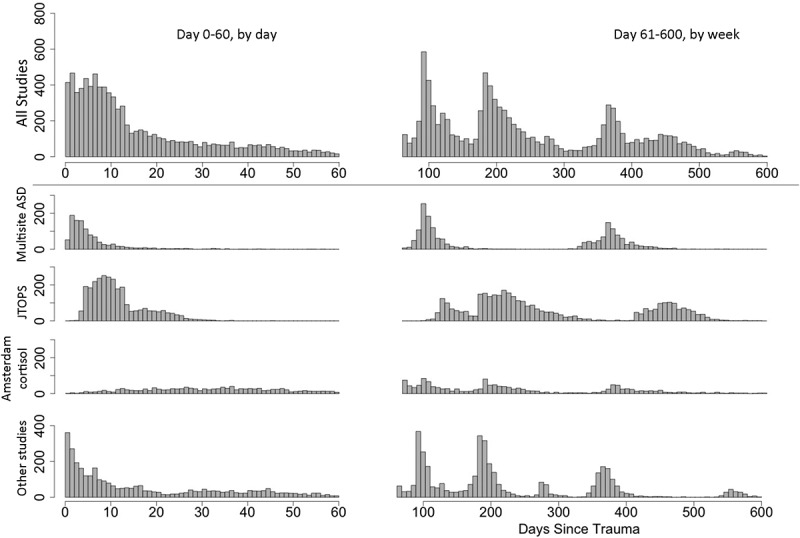
Days since trauma at all assessment time-points. Histogram of days since trauma including all assessment time-points of all instruments in all studies included in the pooled analysis (above the division line), the three largest studies, and other smaller studies (below the division line). The Midwest resilience study was excluded because date information was not available. The number of individuals assessed at a certain day after trauma is represented by the number on the *y*-axis. ASD, acute stress disorder; JTOPS, Jerusalem Trauma Outreach and Prevention Study.

Times of assessments ranged from in-hospital baseline assessment on the day of the traumatic event to 2–3 years later. A total of 175 assessment date variables were found across all studies. Studies differed, however, in their intended assessment timing and actual days since trauma. Some studies had an assessment date input for each instrument or group of instruments (e.g. participants taking different parts of interviews and questionnaires belonging to the same time-point on different days). The days since trauma calculated from these dates were then clustered according to intended time-points from the original studies (e.g. 1 month, 3 months, 1 year). The actual number of days since trauma varied around intended time-points, with some participants seen earlier and many later than scheduled (see Table 4 for details). Figure 1 is a frequency distribution depicting the number of subjects assessed at any given time relative to trauma. The combined data for all studies (except for the Midwest resilience study, owing to a lack of information on assessment dates) are presented in the upper row, and the lower rows show data for the largest individual studies. Figure 1 not only emphasizes the importance of using real time after trauma as a time indicator in pooled data (since data collection periods were often non-overlapping between studies), but also demonstrates that choosing certain time periods in the pooled data may over-sample subjects from certain studies.

**Table 4.** Intended assessment time-points and actual days since trauma (median ± SD) for different studies in the International Consortium to Predict PTSD (ICPP).

Study	<1 month	1 month	3 months	6 months	9 months	1 year	2–3 years
Zurich ICU (Hepp et al., 2008)	11 ± 7			194 ± 18		374 ± 21	1140 ± 41
Hadassah startle (Shalev et al., 2000)	7 ± 4	31 ± 7	101 ± 30				
Jerusalem fMRI (Bonne et al., 2001)	8 ± 4			201 ± 14			
Zurich ward (Jenewein et al., 2009)	4 ± 4			186 ± 16			
Midwest resilience (deRoon-Cassini et al., 2010)	4 ± 4	Date missing	Date missing	Date missing			
Ohio MVA (Irish et al., 2008)	20 ± 6	55 ± 28	104 ± 19	195 ± 22		383 ± 58	
Multisite ASD (Bryant et al., 2008)	4 ± 8		99 ± 22			370 ± 35	
Hadassah cortisol (Shalev et al., 2008)	10 ± 4	36 ± 14	147 ± 44				
JTOPS (Shalev et al., 2012)	9 ± 3	19 ± 5		133 ± 35	220 ± 47	455 ± 103	1060 ± 328
TCOM (Matsuoka et al., 2009a)	2 ± 4	39 ± 10	97 ± 13	193 ± 38	279 ± 18	561 ± 45	1113 ± 44
Amsterdam cortisol (Mouthaan et al., 2014)	24 ± 31	45 ± 28	102 ± 24	207 ± 40		399 ± 70	
Milwaukee ^a^	4 ± 7	54 ± 15		191 ± 17			738 ± 15
Amsterdam oxytocin (van Zuiden et al., 2017)	7 ± 2	46 ± 7	94 ± 11	186 ± 16			
Total	8 ± 13	34 ± 23	100 ± 29	185 ± 45	226 ± 48	410 ± 88	1102 ± 268

^a ^Unpublished at the time of data transfer.

ICU, intensive care unit; fMRI, functional magnetic resonance imaging; MVA, motor vehicle accident; ASD; acute stress disorder; JTOPS, Jerusalem Trauma Outreach and Prevention Study; TCOM, Tachikawa Cohort of Motor Vehicle Accident.

### 3.5. Missing observations

ICPP studies had different rates of attrition (Table 1). Defining loss to follow-up as the absence of follow-up CAPS assessments among those with initial CAPS (*n *= 3909), 667 participants (17%) were lost and 3242 (83%) retained. Participants lost to follow-up did not differ from those retained on baseline CAPS total scores (27.5 ± 25.0 vs 28.6 ± 25.8, respectively, Mann–Whitney U-test *p*-value = 0.46). Nonetheless, future data analyses should assess the nature of loss to follow-up for specific time periods (e.g. 6–9 months, 1–1.5 years) and constructs of interest (e.g. depression, lifetime trauma), and devise ways to palliate for eventual sampling bias (e.g. via selective imputation).

### 3.6. Potential predictors of PTSD

The current work harmonized data for the most consistently measured potential predictors for PTSD. Pre-trauma predictors included gender, education, age, marital status, and prior trauma exposure; peri- and post-trauma predictors include trauma type and acute stress responses. Other risk factors on which fewer studies collected data are not included in the current paper (such as income, pain, and social support); however, these could be analysed in a smaller subset of samples using the same data-processing methods.

### 3.7. PTSD prevalence

Reflecting studies’ inclusion rules, the prevalence of PTSD varied considerably between studies (3.1–61.6% at baseline; 4.3–38.2% on the first follow-up assessment, and 3.8–27.0% on the third assessment) (Table 5). For example, the study with the highest prevalence purposefully recruited participants with a high likelihood of endpoint PTSD, aiming towards evaluating the effect of early interventions (Shalev et al., 2012), whereas other studies recruited liberally among acute care admissions (Wittmann, Moergeli, Martin-Soelch, Znoj, & Schnyder, 2008) without screening for trauma severity and initial symptoms.

**Table 5.** Sample sizes, days since trauma, and PTSD prevalence at different PTSD assessment times.

Study	Assessment 1			Assessment 2			Assessment 3			Assessment 4		
	*N*	DST (mean ± SD)	PTSD (%)	*N*	DST (mean ± SD)	PTSD (%)	*N*	DST (mean ± SD)	PTSD (%)	*N*	DST (mean ± SD)	PTSD (%)
Zurich ICU (Hepp et al., 2008)	121	13.7 ± 6.8	5.8	109	194.1 ± 17.7	4.6	106	373.3 ± 21.5	3.8	90	1142.1 ± 41.0	6.7
Hadassah startle (Shalev et al., 2000)	209	32.8 ± 6.6	21.5	234	107.9 ± 23.4	15.0						
Jerusalem fMRI (Bonne et al., 2001)	43	204.7 ± 14.3	20.9									
Zurich ward (Jenewein et al., 2009)	323	5.0 ± 4.2	3.1	255	188.0 ± 15.7	4.3						
Midwest resilience (deRoon-Cassini et al., 2010)	245	Missing (1 month)	40.4	212	Missing (3 month)	38.2	208	Missing (6 month)				
Ohio MVA (Irish et al., 2008)	278	58.3 ± 15.7	12.6	212	201.3 ± 22.3	8.0	191	392.2 ± 33.4	8.9			
Multisite ASD (Bryant et al., 2008)	1084	7.0 ± 8.3	5.4	931	104.8 ± 18.8	10.3	814	375.3 ± 32.6	9.9			
Hadassah cortisol (Shalev et al., 2008)	131	41.0 ± 13.7	19.1	164	160.1 ± 44.1	21.3						
JTOPS^a^ (Shalev et al., 2012)	1261	9.1 ± 3.0	55.8	932	221.3 ± 39.8	26.2	425	455.9 ± 29.1	21.9			
JTOPS^b^ (Shalev et al., 2012)	735	19.4 ± 5.2	61.6	604	143.7 ± 35.3	26.3	244	342.3 ± 203.9	27.0	336	1053.0 ± 324.8	19.3
TCOM (Matsuoka et al., 2009a)	157	39.3 ± 6.9	6.4	146	196.8 ± 13.7	7.5	78	564.0 ± 24.4	10.3	63	1109.5 ± 24.4	7.9
Amsterdam cortisol (Mouthaan et al., 2014)	620	52.3 ± 27.5	9.4	278	110.1 ± 23.6	6.5	464	216.6 ± 41.1	6.9	405	425.4 ± 70.9	5.9
Milwaukee^c^	211	6.0 ± 7.4	18.5	77	56.0 ± 15.4	27.3	49	194.4 ± 17.5	14.3	47	740.4 ± 15.5	21.3
Amsterdam oxytocin (van Zuiden et al., 2017)	54	6.7 ± 1.8	16.7	48	48.0 ± 6.6	18.8	44	96.3 ± 11.5	9.1	42	191.9 ± 15.5	2.4

^a ^Subgroup from study 9 that only completed telephone interview with the PTSD Symptom Scale.

^b^^ ^Subgroup from study 9 that completed in-person interview with the Clinician Administered PTSD Scale.

^c ^Unpublished at the time of data transfer.

DST, days since trauma; PTSD, post-traumatic stress disorder; ICU, intensive care unit; fMRI, functional magnetic resonance imaging; MVA, motor vehicle accident; ASD, acute stress disorder; JTOPS, Jerusalem Trauma Outreach and Prevention Study; TCOM, Tachikawa Cohort of Motor Vehicle Accident.

## 4. Discussion

The ICPP brought together the largest pooled longitudinal data set of adult civilian trauma survivors to date, containing extensive item-level information on 6254 individuals from 13 studies performed in six different countries. Conceptually, this project hinges on the assumption that PTSD is a robust construct, universally applicable, and therefore amenable to generalization across samples, measures, and data-collection routines (as long as these variations are accounted for). The ICPP effort implies, therefore, that data collected under different circumstances represent subsets of a generic PTSD population. Similar assumptions underlie all current data-pooling enterprises, from genetic consortia such as the Psychiatric Genomics Consortium (http://www.med.unc.edu/pgc/) to the National Institutes of Health data depositories (https://www.nlm.nih.gov/NIHbmic/nih_data_sharing_repositories.html). As such, the ICPP effort, described above, illustrates generic dilemmas and choices made across similar enterprises. The following discussion addresses the specific data features of the ICPP, our approach to pooling and consequent decisions, descriptive results, limitations, and implications for future studies.

### 4.1. Sources of data

Studies in general used analogous recruitment and follow-up templates, adopted similar instruments, and used a common, long-term PTSD outcome. However, participating studies sampled different communities (e.g. communities with differing rates of violent crimes; deRoon-Cassini et al., 2010; Schnyder, Wittmann, Friedrich-Perez, Hepp, & Moergeli, 2008) and applied study-specific inclusion/exclusion criteria (e.g. initial PTSD symptom severity, injury severity, and present and past mental disorders history) (Appendix 1). Pooling across such differences can be seen as an opportunity to create a sample that is more inclusive, more informative of the general link between trauma exposure and PTSD, and less influenced by specific studies’ selection criteria. The combined data set enables a closer examination of studies’ heterogeneities between settings and eras of data collection (1995–2014), and an opportunity to include relatively rare factors in prediction models by recoding and pooling subsets of studies.

### 4.2. Addressing time from trauma

The unique challenge for pooling longitudinal data was to organize the data temporally. As longitudinal cohorts, assessment schedules differed from study to study. They may have been based on theoretical concepts or convenience of sampling. Converting study-defined time-points into real time based on interview dates is the optimal solution to this problem. This allowed us to choose specific time frames after trauma and study the predictive value of factors proximal to trauma on distal outcomes, regardless of the study-specific schedules.

### 4.3. Follow-up timing, duration, and attrition

DSM-IV defined ‘chronic PTSD’ as persisting for more than 3 months. Participating studies’ follow-up assessments extended from 4 months (Shalev et al., 2000, 2008) to over 2 years (Hepp et al., 2008; Shalev et al., 2012), and therefore match the DSM threshold. PTSD persistence beyond a certain time period may become permanent (Marmar et al., 2015): a 6 year follow-up study showed that more than half of the PTSD patients assessed 12 months after trauma continued to meet PTSD diagnosis at a 6 year follow-up (O’Donnell et al., 2016). As such, time to infer PTSD ‘chronicity’ is an important open question, which ICPP data will allow us to approach using the availability of a ‘days since trauma’ measure with a wide range of follow-up times. The choice of specific time ranges will be elaborated upon in relevant individual papers regarding these analyses.

### 4.4. Harmonizing across instruments

One of the major challenges was to organize a great variety of instrumentation. Apart from the common measurements, studies used various instruments to measure different risk factors and different areas of outcomes. Finding common items across different instruments measuring the same construct can maximize obtainable information without absolute equalization. For example, the 17 PTSD symptoms were essential in providing PTSD diagnosis as a major outcome measure in all studies. Nevertheless, higher sensitivity and lower specificity of PSS and PDS compared to CAPS have been reported (Foa & Tolin, 2000; Griffin, Uhlmansiek, Resick, & Mechanic, 2004), and symptoms may be over- or under-reported as a result of different contexts (in-person vs telephone interviews) (Aziz & Kenford, 2004). Harmonizing common items with different wording or scaling usually implies reducing the number of categories to the smallest common denominator, often to dichotomizing. Although the resolution of information is diminished, it is a simple way to maximize the number of studies being analysed. Researchers from a previous pooled analysis [the PTSD after Acute Child Trauma (PACT) data archive] reported similar issues and solutions (Kassam-Adams, Palmieri, Kenardy, & Delahanty, 2011; Kassam-Adams et al., 2012).

When different instruments were used to measure the same disorder, the population effect made it challenging to reach comparable results across the whole sample. The cut-off score for a scale to distinguish psychopathology can vary in different situations and populations (Beekman et al., 1997; Brennan, Worrall-Davies, McMillan, Gilbody, & House, 2010; Cheng & Chan, 2005; Dozois, Dobson, & Ahnberg, 1998; Geisser, Roth, & Robinson, 1997; Hinz & Brähler, 2011; Hiroe et al., 2005; Kugaya, Akechi, Okuyama, Okamura, & Uchitomi, 1998; Lasa, Ayuso-Mateos, Vázquez-Barquero, Díez-Manrique, & Dowrick, 2000; Matsudaira et al., 2009; Wada et al., 2007). Therefore, we could not reliably derive the same diagnosis with different scales in different study populations. Using item response theory to link items from separate instruments to a common scale (Reise & Waller, 2009) may be an approach worth exploring for the next step.

This paper has not included all measures found in the participating studies, especially regarding predictors (e.g. social support, medical/psychiatric history). These parameters were measured in some studies, mostly with study-specific questions, and require further data processing in the relevant studies in order to be pooled.

### 4.5. PTSD outcomes

The project demonstrated that the rates of PTSD outcomes in different studies varied considerably even when study methods were grossly similar. Since each sample was selected according to a series of study-specific criteria, the disparity of PTSD prevalence between studies may be a result of multiple factors in the samples. Inferring PTSD prevalence after a traumatic event in hospital settings simply from a certain cohort study is noticeably inaccurate. Initial symptom severity and prior mental illness can be strong risk factors for later PTSD (Brewin et al., 2000; Ozer et al., 2003). Furthermore, epidemiological studies also reported higher prevalence of PTSD in women, in people living in areas with high community violence, and in victims of interpersonal trauma (Breslau, Chilcoat, Kessler, & Davis, 1999; Breslau et al., 1998; Goldmann et al., 2011; McLean, Asnaani, Litz, & Hofmann, 2011). These population factors, as well as sample sizes and assessment time, may all have contributed to differential PTSD prevalences between studies (Matsuoka, Nishi, Yonemoto, Nakajima, & Kim, 2009b; O’Donnell, Creamer, Bryant, Schnyder, & Shalev, 2003). Pooling these studies together can minimize the effect of study-specific selection and test for results that can be generalizable to the entire trauma population in first responder settings.

### 4.6. Early versus prolonged PTSD

The current literature has not suggested an optimal time for assessing acute or chronic PTSD. One month of symptom duration is needed to meet the DSM criteria for PTSD. However, some researchers choose to evaluate PTSD symptoms much earlier without necessarily diagnosing owing to the difficulty in reaching individuals after their being discharged from hospital (Bonne et al., 2001; Bryant et al., 2008; Hepp et al., 2008). It is noteworthy to recognize that some PTSD symptoms (e.g. insomnia, avoidance) may not manifest as a result of medication or being in a hospital setting. We could not directly observe the impact of early versus late baseline from our results because the time effect is contaminated by the study population effect, as studies adopted different baseline schedules.

### 4.7. Limitations and boundaries to generalization

The ICPP only selected studies from first responder medical settings. The main reason for this is that these settings can best capture people with a distinct, single traumatic event early on. In addition, acute care centres and emergency rooms receive large numbers of potential trauma survivors (Tusche, Smallwood, Bernhardt, & Singer, 2014). Their traumatic events are relatively well documented. Since we did not include any studies of people with chronic or repetitive trauma, such as refugee or family violence studies, and military or war-zone studies, the results of this study may not be generalizable to these trauma populations. Nevertheless, the methods explored in this study may inform studies in other settings. Another limitation of this study is that all PTSD measures are under the DSM-IV/International Statistical Classification of Diseases and Related Health Problems, 10th Revision (ICD-10) system. DSM-5 has additional symptom criteria that were not measured in the original studies. Future work might substantiate the effects and the implications of shifting diagnostic templates (Hoge, Riviere, Wilk, Herrell, & Weathers, 2014).

### 4.8. Implication for future studies

Publicly funded projects are moving towards an era of data sharing, and the ability to utilize shared data in an effective way can profoundly deepen the understanding of individual research findings. Although it is much more time-consuming to pool individual data than summary results, it provides a harmonized data set which can be subsequently used to calculate predictive probabilities, discover symptom trajectories, and conduct analyses that were not involved in the original studies (e.g. random forest). The lessons we learned from pooling ICPP data have a few implications for future efforts. First, before planning prospective studies, it may be helpful for researchers to expect the usage of their data in future pooling and to facilitate the process by documenting study-specific features in detail. Using common data elements, such as the PhenX toolkit (Hamilton et al., 2011), is highly recommended. This practice will facilitate future pooled analysis and maximize poolable information. Secondly, data processing and quality control require careful planning and laborious work. Resources need to be sufficiently allocated in these areas for pooled data projects. Most importantly, future studies should be encouraged to produce more accessible data, and conduct more efficient and informative large-scale analyses. The work from the ICPP has made great efforts in advancing the current field of prediction in PTSD, strengthening the empirical database and opening gateways for more robust clinical prediction tools leading to targeted intervention.

## 5. Conclusions

As this work has shown, pooling different data sets at the item level is an important step towards more robust and generalizable findings in the population. Although pooling longitudinal data is far from simple and requires content expertise, this method supports innovative data analyses which might not have been conducted in original studies and promotes results from a heterogeneous population that is less constricted by study selection criteria. The crucial question in pooling is how to acquire data from various sources and maximize obtainable information. While few variables are uniformly annotated across studies, most others require informed decisions as to their harmonization and formatting in the pooled data set. In this work, relative homogeneity and clarity of a few reliably poolable variables (e.g. gender, age, PTSD severity and status, time from trauma) constituted a precondition for pooling. Many variables measuring the same constructs (e.g. trauma type, education, marital status, prior trauma) could be recoded and organized, and ultimately constituted an informative ensemble. Several instruments were used to capture a certain construct (e.g. depression). Using such information requires cross-instrument harmonization, which constrains the depth of information available for subsequent analyses. Ultimately, much of the present work amounts to defining the resolution within which a data set can be questioned, and has implications for future studies pooling item-level data from data repositories.

## Figures and Tables

**Figure A1.**
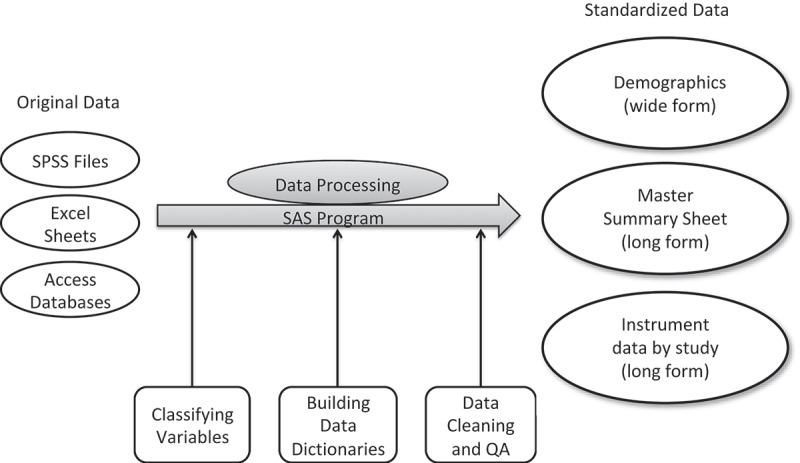
Data processing procedures.

**Figure A2.**
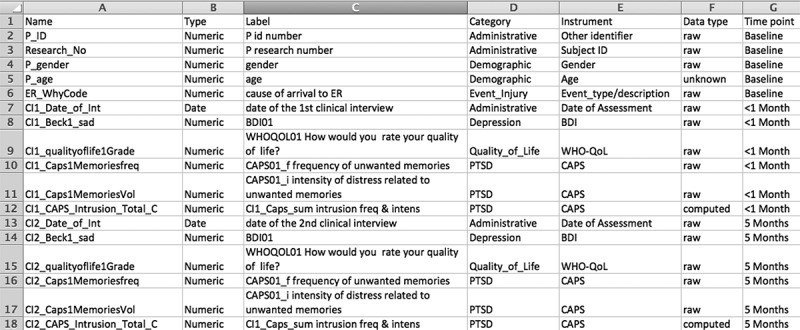
Variable classification example.

## Appendix 1. Study recruitment features and inclusion/exclusion criteria

**Table:**

Study	Zurich ICU	Hadassah startle	Jerusalem fMRI	Zurich ward	Midwest resilience	Ohio MVA	Multisite ASD	Hadassah cortisol	JTOPS	TCOM	Amsterdam cortisol	Milwaukee	Amsterdam oxytocin	Studies with criterion (*n*)
**Setting**														
ER admissions		X	X				X	X	X	X	X		X	8
Trauma ward/ICU admissions	X			X	X	X						X		5
**Intervention study**														
Not intervention study	X	X	X	X	X	X	X	X		X	X	X		11
Control group of intervention study													X	1
Active treatment (in part of the sample)									X					1
**Inclusion**														
Age minimum (years)	18	16	18	18	18	18	16		18	18	18		18	11
Age maximum (years)	70	65	65	65			70		70	69			65	8
Language requirement	German			German; Italian; Spanish; Portuguese; Serbo-Croatian; Turkish; Albanian	English	English	English		Hebrew; Arabic; English	Japanese	Dutch		Dutch; English	9
A1 criterion		X	X						X		X		X	5
Admission length minimum (hours)				32	48		24							3
A2 criterion			X						X					2
Motor vehicle accident victim						X				X				2
Life-threatening or critical condition	X									X				2
Traumatic injury (soft tissue, internal, orthopaedic)					X									1
TSQ ≥ 5 and/or PDI ≥ 17													X	1
**Exclusion**														
*Injury severity criteria*														
GCS minimum	9			9		14					13		13	5
Severe head/brain injury				X			X			X	X			4
Head injury: LOC maximum (min)		Any LOC		15				Any LOC						3
Moderate head/brain injury							X				X			2
Admission length maximum (h)									168					1
ISS minimum	10													1
Unconscious upon admission									X					1
Injury requires (major) surgery								X						1
Intravenous catheter inserted								X						1
Burn injury		X												1
Develops life-threatening medical illness		X												1
Spinal cord injury with neurological deficit					X									1
*Cognitive criteria*														
MMSE minimum					21	25				24				3
Cognitive impairment							X						X	2
*Event criteria*														
Injury a result of self-harm	X			X	X						X			4
Injury resulting from intentional harm by others	X			X										2
Injury occurred when intoxicated								X						1
*Health criteria*														
Medical or surgical conditions that interfered with participation	X			X	X				X	X				5
Currently psychotic (or bipolar)		X					X			X	X		X	5
Currently suicidal							X			X			X	3
Current drug abuse		X								X			X	3
Current alcohol abuse		X											X	2
Organic brain condition											X		X	2
Pregnancy								X					X	2
Pre-existing chronic PTSD								X					X	2
History of neurological disorder			X										X	2
History of psychosis		X	X											2
History of alcohol abuse		X	X											2
History of drug abuse		X	X											2
Current severe depressive disorder													X	1
Current severe personality disorder													X	1
Current physical illness not related to trauma	X													1
Serious dissociation										X				1
Chronic endocrine disorder								X						1
Chronic disease (high BO, arthritis, diabetes)													X	1
Pre-existing non-trauma-related mental health problems	X													1
Mental retardation													X	1
History of PTSD			X											1
History of epilepsy										X				1
*Practical criteria*														
Distance from site or residency criterion						X	X		X	X				4
Ongoing victimization		X						X					X	3
Under police guard/incarcerated							X							1
Counterindication for oxytocin													X	1
Experienced other TE during 6 month follow-up			X											1
Substance abuse during 6 month follow-up			X											1

ICU, intensive care unit; fMRI, functional magnetic resonance imaging; MVA, motor vehicle accident; ASD, acute stress disorder; JTOPS, Jerusalem Trauma Outreach and Prevention Study; TCOM, Tachikawa Cohort of Motor Vehicle Accident; ER, emergency room; TSQ, Trauma Screening Questionnaire; PDI, Peritraumatic Distress Inventory; GCS, Glasgow Coma Scale; LOC, loss of consciousness; ISS, Injury Severity Score; MMSE, Mini-Mental State Examination; PTSD, post-traumatic stress disorder; BP, blood pressure TE, traumatic event.

## Appendix 2. Data-Set Creation, Cleaning and Quality Assurance Procedures of the International Consortium to Predict PTSD (ICPP)

### Overview

The International Consortium to Predict PTSD (ICPP) seeks to develop versatile tools for predicting post-traumatic stress disorder (PTSD) by pooling longitudinal data sets measuring early trauma aftermath. To date, the ICPP has included original data sets from 13 longitudinal studies conducted between 1998 and 2014. These studies were conducted in a variety of nations across several continents using different languages. In these studies, primary investigators obtained a baseline assessment shortly after trauma exposure, followed respondents for at least 1 month, assessed both PTSD diagnosis and the severity of PTSD symptoms, and provided item-level data. A wide variety of instruments evaluated an extensive array of trauma-related outcomes.

Participants were assessed at different time-points, including emergency room, and 1 week, 1 month, 3 months, 4 months, 5 months, 6 months, 9 months, 12 months, 18 months, 2 years, and 3 years after trauma. Each studied adopted two to five time-points among them, with some overlap across studies. We examined the assessment dates to calculate true time from trauma in order to standardize the indicator of follow-up time.

Our goal in this project is to construct standardized item-level data sets that will be of significant value when used in analyses leading towards advances in predicting and treating PTSD.

We created a demographic sheet with all subjects from the original data files. Their age, gender, education, marital status, trauma type, and prior trauma information was included. We also created a data summary sheet with the number of assessments for each instrument, and days since trauma at different assessment points. These two documents serve as the anchor for merging different instruments once they are cleaned and processed into standardized format (Figure A1).

### Original data

Data were received in a wide variety of formats, including SPSS files, Excel spreadsheets, and Access databases. Our staff used the SAS language to process all data, with the ultimate outcome being labelled SPSS files, or any other formats that statisticians request (e.g. CSV).

Most of the data sets contained all data for a subject in one record (wide form), i.e. data points for all interviews at every time-point, with all dates and demographic information as well. In some studies, however, data were transferred in a number of separate data files, each containing one time-point or one/several instrument(s). As a result, variable names were not the same in the data for any instrument across studies or across time-points.

### Classifying variables

This is the first step towards understanding and processing raw data from original files. A research associate and a post-doctoral student looked through all variables in each original data set, identified which instrument each variable belonged to, and classified instruments into different categories for sorting. Figure A2 shows an example of how variables were classified in a study.

### Data dictionaries

Before a data set can be created, a data dictionary has to be created for its corresponding instrument. Working in conjunction with the owner of each data set, we ascertained the names and time-points of the variables for each instrument in a study’s data set. We used our own naming convention for the data points in each instrument, with each variable name consisting of between six and eight characters. The variable name combines the acronym for the instrument with the sequential number of the variable in the instrument. For example, the first variable in the Hospital Anxiety and Depression Scale data set is named HADS01.

The data dictionary contains useful information pertaining to each variable, including:

- master variable name
- descriptive information used for labelling
- data type (i.e. integer, text, date)
- valid response codes (e.g. 0–4)
- valid response descriptions (descriptions for each valid response code).

### SAS programs

With the data dictionary providing a blueprint, a unique SAS program was written to transform all the data for an instrument in a study into a cohesive data set. The data set contains one record for each subject at every time-point for which data were collected for the instrument. If no responses were elicited for the instrument from a subject on any given interview date, the resulting blank record was not included in the final data set.

### Master Summary sheet

The Master Summary sheet is the most important ‘road map’ guiding the creation of final data sets for analysis. It follows the long form, which means that the number of records per subject equals the number of time-points when the subject was seen. We have structured the data sets from each instrument to contain a group of standard variables including a subject identifier that is unique for every subject in the database, the date of the traumatic event, the date of the interview with the subject, and a calculated variable of the number of days that have elapsed since the traumatic event (at the time of each interview). There is also an interview time-point description variable that fits the time-point into a range of time, e.g. baseline, < 1 month, 1–3 months, 3–6 months. The time description is consistent across studies. The Midwest study only had the date of the first interview, and the days since trauma at the first interview. We calculated the trauma date accordingly, and added 30, 90, and 180 days to the first interview date to generate subsequent days since trauma. For this study, days since trauma only serve as indicators to facilitate merging and do not represent real time since trauma.

### Demographics sheet

The demographics sheet contains age, gender, level of formal education, marital status, and other available baseline information, such as trauma type and prior trauma, when available. Each subject has only one record in the data set (wide form). This file contains all subjects with any data from all original data files collected. It provides information on the number of subjects and some essential baseline information as potential predictors.

### Standard instrument data

Standard data sets were created for an instrument for each study that contained data for that instrument. Data were cleaned and then given a thorough quality assurance check. A merged instrument data set can be created containing all records from all standard data sets with this instrument. Using the Clinician Administered PTSD scale (CAPS) data set as an example, it was then possible to merge all variables to each record. Our master CAPS data set contains 3952 subjects and almost 10,000 records.

### Cleaning data

We look to clean data as quickly and accurately as possible from the moment we create a data set. Any actions taken to clean data are always thoroughly documented.

One good example of our data cleaning effort is the days since traumatic event (DST) variable. We first correct data-entry errors. The interview date should never precede the date of the traumatic event. In such instances, the DST value will be a negative number. We can look for such values and make a quick determination as to whether a reasonable assumption can be made regarding the error. In the majority of such cases, the interview occurred shortly after the beginning of a new calendar year and the year value of the interview date was inadvertently that of the prior year. Correction of these errors can then be made in the data-set creation program. In circumstances when such assumptions of data-entry errors cannot be made, we write back to data owners to track back their original records, such as paper logs. When these records are not available, we make the variable missing.

For data points that have a set of acceptable, valid responses, we write out-of-range data checks in our data cleaning programs to find values that do not match up with a corresponding valid response description. For these records, we will contact the owner of that study’s data to see whether an acceptable explanation can be provided for the out-of-range value. If so, we are then usually able to programmatically map the data point to a valid response.

### Quality assurance

Our data quality-assurance process begins with the transformation of the original data set into a usable file for processing, and does not end until we feel the final data sets are as clean and accurate as possible. During the development and data-set creation processes, our programmers continuously browse the intermediate files with which they work to look for any potential problems in the data.

The data sets can be compared to living entities, in that they are created, and often grow and expand as they are used in analysis, and sometimes merge additional/derived variables and calculated scores into records. At each point along the way, statisticians, research coordinators and programmers are looking closely at the files with which they work to guarantee that their quality is of the highest standard. Potential problems are discussed as a group, and solutions are sought with input from everyone involved.

We continue to receive data from additional studies and researchers, and continue to enlarge our longitudinal PTSD database following the above procedures.
